# Developing Clinically Relevant Dissolution Specifications (CRDSs) for Oral Drug Products: Virtual Webinar Series

**DOI:** 10.3390/pharmaceutics14051010

**Published:** 2022-05-07

**Authors:** Mark McAllister, Talia Flanagan, Susan Cole, Andreas Abend, Evangelos Kotzagiorgis, Jobst Limberg, Heather Mead, Victor Mangas-Sanjuan, Paul A. Dickinson, Andrea Moir, Xavier Pepin, Diansong Zhou, Christophe Tistaert, Aristides Dokoumetzidis, Om Anand, Maxime Le Merdy, David B. Turner, Brendan T. Griffin, Adam Darwich, Jennifer Dressman, Claire Mackie

**Affiliations:** 1 Pfizer WWRD, Sandwich, Kent CT13 9NJ, UK; mark.mcallister@pfizer.com; 2 UCB Pharma SA, 1420 Braine l’Alleud, Belgium; talia.flanagan@ucb.com; 3 Medicines & Healthcare Products Regulatory Agency, London E14 4PU, UK; susan.cole@mhra.gov.uk; 4 Development Sciences and Clinical Supply, Merck & Co., Inc., Rahway, NJ 07033, USA; andreas_abend@merck.com; 5 European Medicines Agency, 1083 Amsterdam, The Netherlands; evangelos.kotzagiorgis@ema.europa.eu; 6 Federal Institute for Drugs and Medical Devices (BfArM), 53175 Bonn, Germany; jobst.limberg@bfarm.de; 7 AstraZeneca UK Limited, Macclesfield SK10 2NA, UK; heather.mead@astrazeneca.com (H.M.); andrea.moir@astrazeneca.com (A.M.); xavier.pepin@simulations-plus.com (X.P.); 8 Department of Pharmacy and Pharmaceutical Technology and Parasitology, University of Valencia, E46100 Burjassot, Spain; victor.mangas@uv.es; 9 Interuniversity Research Institute for Molecular Recognition and Technological Development, Polytechnic University of Valencia, 46010 Valencia, Spain; 10 SEDA Pharmaceutical Development Sciences, Stockport SKB 3GX, UK; paul.dickinson@sedapds.com; 11 Simulations Plus, Inc., Lancaster, CA 93534, USA; maxime.lemerdy@simulations-plus.com; 12 AstraZeneca Boston, Boston, MA 02451, USA; diansong.zhou@astrazeneca.com; 13 Janssen Research and Development, 2340 Beerse, Belgium; ctistaer@its.jnj.com; 14 Department of Pharmacy, National and Kapodistrian University of Athens, 15784 Athens, Greece; adokoum@pharm.uoa.gr; 15 Division of Biopharmaceutics, Office of New Drug Products, Office of Pharmaceutical Quality (OPQ), Centre for Drug Evaluation and Research, Food and Drug Administration (FDA), Silver Spring, MD 20903, USA; om.anand@fda.hhs.gov; 16 Certara UK, Simcyp Division, Sheffield S1 2BJ, UK; david.turner@certara.com; 17 School of Pharmacy, University College Cork, T12 YT20 Cork, Ireland; brendan.griffin@ucc.ie; 18 Division of Health Informatics and Logistics, KTH Royal Institute of Technology, SE-164 40 Stockholm, Sweden; darwich@kth.se; 19 Fraunhofer Institute of Translational Medicine and Pharmacology, 60528 Frankfurt am Main, Germany; jdressman@em.uni-frankfurt.de

**Keywords:** oral drug products, clinically relevant dissolution specifications, PBBM, product performance, biorelevant dissolution

## Abstract

A webinar series that was organised by the Academy of Pharmaceutical Sciences Biopharmaceutics focus group in 2021 focused on the challenges of developing clinically relevant dissolution specifications (CRDSs) for oral drug products. Industrial scientists, together with regulatory and academic scientists, came together through a series of six webinars, to discuss progress in the field, emerging trends, and areas for continued collaboration and harmonisation. Each webinar also hosted a Q&A session where participants could discuss the shared topic and information. Although it was clear from the presentations and Q&A sessions that we continue to make progress in the field of CRDSs and the utility/success of PBBM, there is also a need to continue the momentum and dialogue between the industry and regulators. Five key areas were identified which require further discussion and harmonisation.

## 1. Introduction

From January 2021 to June 2021, a virtual webinar series entitled ‘Developing Clinically Relevant Dissolution Specifications for Oral Drug products’ was organised by the Academy of Pharmaceutical Sciences (APS) Biopharmaceutics Science focus group with speakers including colleagues from the industry, academia, and regulatory bodies. A summary of a previous workshop organised by APS on this topic was published in 2020 [1].

In the period since the first workshop, there has been significant activity in this area, particularly with the application of physiologically based biopharmaceutics modelling (PBBM), to support the development of CRDSs. In addition to publications describing the use of PBBM to support product development [2,3,4,5,6] and continued dialogue between academic, industrial, and regulatory scientists [7,8,9], the FDA has published a Draft Guidance document on the use of physiologically based pharmacokinetic (PBPK) analyses to support oral drug product development, manufacturing changes, and controls [10]. The aim of the 2021 webinar series was to continue the dialogue from the previous workshop and share and discuss progress and challenges through scientific presentations and Q&A sessions. The webinars reviewed the current state of scientific progress in the field and emerging trends, identifying areas where industry and regulators can engage to further harmonise and enable the future development of CRDSs. The webinar series included six webinars covering the following topics:

1. Introduction to CRDSs—the ‘what’ and the ‘why’;
2. Introduction to PBPK/PBBM Modelling—the ‘when’ and the ‘how’;
3. How to develop CRDSs, including case studies from industry;
4. Overview of global regulatory trends within CRDSs, including progress, challenges, and emerging opportunities;
5. Future developments with PBBM/PBPK software packages;
6. Emerging opportunities within PBPK/PBBM modelling to support CRDSs, including new research areas.

The Q&A sessions at each webinar provided colleagues/delegates with an opportunity to discuss the information and content shared and highlight the major themes which could be taken forwards both to advance this key area and to shape the next series of meetings on this topic.

## 2. Summary of Webinars

### 2.1. Webinar 1: Clinically Relevant Dissolution Specifications: Why, What, How, and When? (Paul A. Dickinson and Andreas Abend)

#### 2.1.1. The Why and What (Paul Dickinson)

In other industries that serve the public, it is routine for the design, build, and test process to result in an understanding of what needs to be measured to ensure the safe and effective performance of the product. These tests and associated acceptance ranges (i.e., specifications) measure *critical quality attributes* (CQAs), and they are clear-cut and unmistakable, such as the engineering tolerance on a certain component. This should also be the expectation for drug products such that their suitability is supported by clear-cut and unmistakable specifications which ensure consistent performance in patients. Some pharmaceutical product specifications are undisputed and well-accepted such as:

- Identity (safety and efficacy);
- Assay (safety and efficacy);
- Related substances/degradants (safety).

For these specifications, methods can be developed that can accurately measure these CQAs and different methods (e.g., analytical tests probing the same attribute) will give the ‘same’ result if suitably developed. The term ‘clinical relevance’ was introduced in 2008 [11] and subsequently became clinically relevant drug product specifications to suggest that there is a direct link between drug product specifications and clinical outcomes. The acceptance criteria for assay and impurity specifications are based on internationally accepted ranges (cite ICH guidance here) or compendia (assay) and not on actual ranges studied in clinical trials.

From a patient perspective, the rate and extent of drug release in the body (i.e., in vivo dissolution) resulting in consistent in vivo performance (e.g., bioperformance) is without a doubt a critical quality attribute. Measuring the rate and extent of drug release in vivo is impractical, and thus, this CQA is assessed via in vitro dissolution. Currently, highly sophisticated dissolution apparatus, with tight instrument specifications, are used to assess the rate and extent of drug release, although they are far from representing the complexity of the human GI tract. However, the equipment that is internationally recognised by regulatory agencies as acceptable to probe in vivo dissolution is usually more or less a modification of a beaker with a stirring device. For each product, experimental conditions, such as the dissolution media and level of agitation under which the product will dissolve, are regulatory requirements and, therefore, must be developed and used for product release. Unlike the specifications for impurity, assay, and identity, performing dissolution testing under different conditions will usually result in different outcomes. Therefore, the choice of the dissolution equipment, the operating conditions (i.e., volume and agitation), and the dissolution media conditions are key challenges in product development. The purpose of the dissolution test is elegantly expressed in current significant regulatory guidance as having the following features:

1. An important surrogate of clinical performance;
2. A routine test of product quality.

*‘Drug absorption from a solid dosage form after oral administration depends on the release of the drug substance from the drug product, the dissolution or solubilization of the drug under physiological conditions, and the permeability across the gastrointestinal tract. Because of the critical nature of the first two of these steps, in vitro dissolution may be relevant to the prediction of in vivo performance. Based on this general consideration, in vitro dissolution tests for immediate release solid oral dosage forms, such as tablets and capsules, are used to (1) assess the lot-to-lot quality of a drug product; (2) guide development of new formulations; and (3) ensure continuing product quality and performance after certain changes, such as changes in the formulation, the manufacturing process, the site of manufacture, and the scale-up of the manufacturing process’*

[12]

*‘A dissolution procedure intended to be used as a routine control test for immediate release drug products should be robust, reproducible and discriminatory in order to assure a consistent product quality and to detect product quality attributes, which, if altered, may affect the in vivo performance’*

[13].

This mixed role of covering both aspects of in vivo performance and quality testing leads to challenges experienced in defining/accepting a dissolution specification for routine product release by regulatory agencies worldwide. Particularly when considering quality aspects, the dissolution specification is often seen by regulatory agencies as a ‘global’ test that should be sensitive towards any manufacturing variable (drug substance, ingredients, and process conditions) that could impact in vivo performance. This often leads to overly sensitive dissolution conditions towards manufacturing conditions which then require tight process controls. To ensure every product batch released to patients performs according to the product label, regulatory agencies typically allow dissolution specifications only based on the release data from clinical trial materials used in pivotal trials—unless justified by additional in vivo data. On the other hand, companies often develop alternative and complex dissolution methods to guide drug formulation and process development. 

These methods are developed to assess the impact of materials attributes that could impact in vivo performance and to support the development of robust manufacturing control strategies. Companies often refine these methods based on in vivo data collected throughout development and use the insights gained from these methods to guide the development of a quality control specification suitable for day-to-day release testing in a QC environment. The conundrum for industry and regulatory agencies is that the regulatorily approved dissolution specification may not be sensitive towards materials attributes that impact in vivo performance. Companies, therefore, develop control strategies that are independent of a dissolution specification to assure appropriate product performance. This often then renders the QC dissolution specification for immediate release oral dosage forms meaningless although dissolution specifications are a regulatory requirement. Unfortunately, global acceptance of QC dissolution specifications requires ‘sensitivity’ towards product materials attributes or ‘critical’ processing parameters which then often leads to the development of overly sensitive specifications and unnecessarily tight process controls and potentially discard of product. To avoid this dilemma, the FDA [10], and EMA [13] encourage companies to support the dissolution specification by studying process variants with different in vitro release profiles, representing a range of critical material attributes that may impact in vivo performance, and to link the results to clinical trial materials used to establish the safety and efficacy of the drug. Thus, a clear link between the clinical performance of the drug, critical material attributes, and dissolution specifications can be made, and the method is deemed ‘clinically relevant’. Although the focus of CRDSs is generally on oral drug products, these concepts equally apply to all drug products for which there is a release or dissolution step.

When first considering CRDSs, superficially, it can give the impression that it is all about clinical studies on drug product variants with different dissolution profiles and dissolution tests. CRDSs, however, should be viewed as the outcome of efforts to build a knowledge base that allows an understanding of clinical drug product performance to be developed and then allow the level of risk associated with the test and control of clinical quality to be described such that the performance (quality) of product delivered to the patient is acceptable. This, by default, involves many disciplines.

It is, therefore, suggested that a systems approach to developing the understanding required to underpin CRDSs is necessary and that good biopharmaceutics risk understanding/ability to describe the clinical performance is essential. This implies that these aspects should be considered right from the start of clinical development (CRDS is often seen as a later-phase activity).

BioRAM is a useful framework to implement systems thinking throughout development and highlights the concept of an early QTPP [14,15,16]. It centres on answering critical questions, moving quickly to decision points to ensure that patient-centric drug products are developed, and generating the necessary knowledge and data to ensure appropriate drug product quality can be maintained, both in in pivotal studies and following launch. Following the roadmap including building an early QTPP and using the scoring grid facilitates this process, resulting in CRDSs.

#### 2.1.2. The How and When (Andreas Abend)

Different approaches exist for the development of CRDSs for solid oral drug products. The applicant needs to decide if, how, and when establishing a link between in vitro dissolution and in vivo performance is critical to guide drug product development, the release of product batches in the clinical trial phase, and/or for the commercial product and to enable efficient product lifecycle management. Rational decision making should be based on a biopharmaceutics risk assessment, which identifies if variability in in vivo dissolution, because of manufacturing changes, has an unacceptable impact on product performance; therefore, it needs to be controlled via in vitro specifications.

For most immediate-release tablets containing BCS 1 or BCS 3 compounds, in vivo product performance is usually robust against manufacturing variations, and a dissolution specification to ensure consistent product performance is often unnecessary. On the other hand, controlling critical material attributes and critical process parameters that may impact bioperformance for solid oral dosage forms containing poorly soluble drug substances may be critical to ensure product quality. As a result of the complex gastrointestinal environment a drug encounters after oral administration, establishing an in vitro dissolution method with adequate sensitivity to detect unacceptable in vivo behaviour is challenging. Without in vivo studies aimed at bridging the impact of product variations on in vitro dissolution and in vivo performance, the clinical relevance of an in vitro dissolution method may be unknown.

Product developers can often choose between traditional clinical PK studies aimed at establishing an in vitro in vivo correlation or BE between two formulations, or they can attempt to build a mechanistic understanding of in vivo dissolution via physiologically based biopharmaceutics modelling. In either case, the clinical relevance of dissolution specifications can be established such that only a product with acceptable bioperformance is released to the patient. In addition, a clinically relevant dissolution method provides an in vitro safe space that can be used to justify (moderate) post-approval changes. Using a dissolution safe space is superior to currently required dissolution profile comparisons and the f2 metric since these experimental conditions and the questionable f2 similarity acceptance criteria have no proven ability to indicate unacceptable changes in in vivo behaviour.

### 2.2. Webinar 2: Introduction to PBPK/PBBM: How to Build a PBBM Model and Why? (Andrea Moir and Sue Cole)

Several approaches to absorption model development can be adopted—from empirical compartmental modelling to more mechanistic physiologically based pharmaco-kinetic modelling (PBBK) and physiologically based biopharmaceutics modelling (PBBM). Compartmental models provide a mathematical description of absorption, distribution, metabolism, and elimination (ADME) using first-order rate constants and virtual volumes, with the PK data fitted to one, two, or three compartments. By comparison, multicompartmental PBPK models incorporate physiological properties, such as tissue volumes and compositions and the impact of enzymes, transporters, fluid dynamics, and transit times. Building further on this, the aim of a PBBM is to achieve a mechanistic model that describes the drug substance, drug product, and physiological properties and processes involved from the point of administration of the drug, (with particular consideration here to solid, oral dosage forms) to absorption and subsequent distribution, metabolism, and excretion [17].

PBBM can be used in the justification and setting of clinically relevant dissolution specifications through the establishment of a dissolution safe space, within which in vitro dissolution changes have no detrimental impact on in vivo performance, or by establishing an edge of failure beyond which changes in in vitro dissolution have an unacceptable impact on PK. Coupling such approaches with virtual bioequivalence analysis (VBE) [18,19] can be used to obtain biowaivers for generic products or used by originators during product development, for example, to assess impact after scale-up and post-approval changes (SUPAC), or changes in supplier or grade of excipients. Best practice guidelines for wider PBPK applications beyond the biopharmaceutic area have been published [20], and there is recent interest in the setting of best practice guidelines for PBPK modelling in the biopharmaceutic area [10,21].

Regulatory guidelines are available to aid in PBPK/PBBM model development and evaluation to support models used in regulatory submissions. The relevant guidelines are as follows:

- Guideline on the pharmacokinetic and clinical evaluation, modified release dosage forms 2014 [22];
- Guideline on the reporting of physiologically based pharmacokinetic (PBPK) modelling and simulation 2018 [23];
- FDA Guidance on ‘Physiologically Based Pharmacokinetic Analyses—Format and Content Guidance for Industry’ [24];
- The use of physiologically based pharmacokinetic analyses—biopharmaceutics applications for oral drug product development, manufacturing changes, and controls [10].

The EMA PBPK guideline on modelling and simulation includes extensive detail on model evaluation and qualification as two appendices, the requirements for these depend on the model impact.

An Impact framework has been used for some time in regulatory consideration of models [25], and requirements for model evaluation increase with the extent of the impact of the model. Models with high impact, e.g., those replacing clinical studies, will require a high level of evaluation and qualification. Qualification is the term adopted by the EMA and is defined as ‘The process of establishing confidence in a PBPK platform to simulate a certain scenario, in a specific context, based on scientific principles and ability to predict a large dataset of independent data thereby showing the platforms ability to predict a certain purpose’. This is directly related to the use of the model, e.g., DDIs, paediatrics, or CRDSs. Some guidance is given on what should be included in the qualification dataset for a high-impact application of a model.

The guidance also gives recommendations on the platform and drug model evaluation. It is recommended that the predictive performance of the model should be rigorously assessed, this is defined as ‘*the basis of how well important characteristics of the drug model has been tested against in vivo PK data and whether adequate sensitivity and uncertainty analyses have been conducted to support the model’s ability to provide reliable predictions*’. Sensitivity analyses provide important knowledge of the uncertainty in the model and hence inform the level of confidence. The model should be tested against clinical data usually for a range of doses and conditions, e.g., fed and fasted. There are no criteria for the goodness of fit, but instead, this is proposed to be considered in terms of the known exposure response for efficacy and safety. For biopharmaceutical applications, a confidence interval such as those used for bioequivalence testing may be indicated. It is also important to determine if the variability in clinical data is adequately captured by the model.

In Nov 2019, the FDA held a workshop to discuss the development of best practices in physiologically based pharmacokinetic modelling to support clinical pharmacology regulatory decision making [26]. At this workshop, the credibility assessment framework in model-informed drug development was introduced [27]. The framework consists of the following five key concepts:

1. Question of interest;
2. Context of use—how is the model used to answer the question?;
3. Assess model risk—what weight does the model bring to the decision? Additionally, what would be the consequence of a wrong decision?;
4. Establish risk-informed credibility assessment—this needs to be commensurate with the risk and requires validation and verification activities;
5. Model credibility assessment—considered against the context of use. This approach is consistent with that recommended by EMA Guidance. To date, there are no examples in the literature of the application of this framework to PBBM, but it is referred to in the recent draft FDA guideline ‘The Use of Physiologically Based Pharmacokinetic Analyses’ [10].

**Figure 1:**
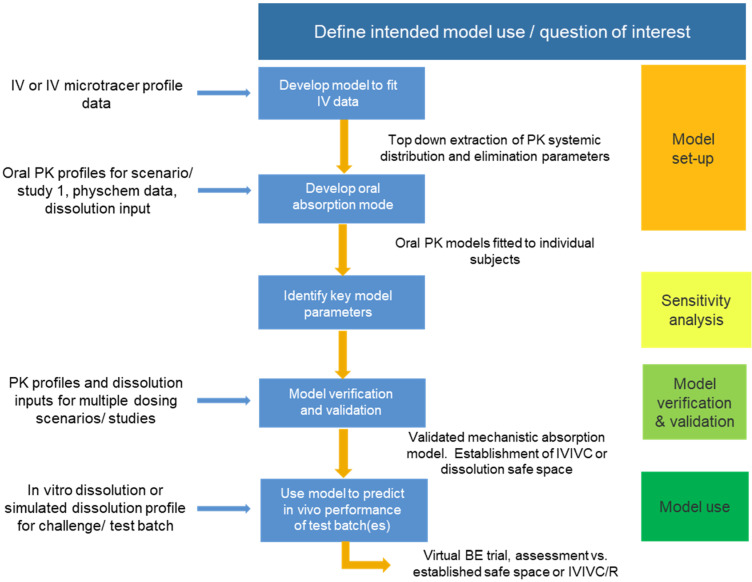
Schematic of approach for PBBM model development, validation, and use.

Taking into consideration the regulatory guidelines detailed above, a schematic approach to model development, validation, and use is presented in Figure 1.

The validation of the model must use a separate dataset from that of the model setup and should comprise variants representative of the commercial formulation. The validation batches may encompass variations in critical material attributes (CMAs), such as DS particle size or polymer grade/molecular weight, and critical process parameters (CPPs), which are pertinent to the dissolution specification.

The reference and variant batches should be evaluated in the clinic using a crossover design. The inclusion of an IV arm or IV microdose enables a top-down extraction of distribution and elimination parameters. PK sampling times should be selected to enable the gastric emptying and absorption phases to be well defined. An enhanced understanding of the gastrointestinal (GI) physiology can be gained through the inclusion of markers in the clinical study, providing information on the in vivo conditions for each subject in which dissolution would occur [28,29].

Consideration should be given to how best to integrate in vitro dissolution data into the PBBM. Approaches include direct input of % dissolution vs. time or fitting a Z factor, Weibull function, or product particle size distribution (P-PSD) into the dissolution profile. The choice of approach will depend on formulation type (immediate or modified release) and whether the dissolution rate or extent is dependent on the media pH or volume [17].

PBBM has successfully been used to support the dissolution specification for ZURAMPIC (lesinurad) tablets [30]. The model was established using an IV microdose and reference tablet batch and then validated using dissolution and clinical data for a bioinequivalent batch. P-PSD, Weibull, and Z-factor approaches were explored for the integration of dissolution data; among them, P-PSD (validated across a range of dissolution media) was the only option that enabled bioinequivalence to be reproduced. The model was then used to predict the likely in vivo performance of tablet batches at the limits of the dissolution specification. A comparison of AUC and C_max_ values relative to the reference tablet batch indicated that the batches are likely to be bioequivalent in an appropriately powered clinical study. Finally, a virtual batch dissolution profile was used as an input in the model to define the boundaries of failure of the dissolution safe space.

### 2.3. Webinar 3: How to Develop CRDSs including Case Studies from the Industry (Xavier Pepin, Diansong Zhou, and Christophe Tistaert)

#### 2.3.1. Case Study 1—PBPK Application to Evaluate Acalabrutinib Absorption and Drug Substance PSD Safe Space Size (Xavier Pepin and Diansong Zhou)

Physiologically based biopharmaceutics models (PBBMs) can be used to define acceptable product specifications in terms of critical material attributes or process parameters. The integration of drug product dissolution and drug substance particle size in the PBPK tool was evaluated for Simcyp using acalabrutinib, a BCS Class 2 drug, as an example. Two approaches were used: a mechanistic diffusion layer model (DLM) scaling factor applied to measured drug substance particle size to fit product dissolution data or a product particle size distribution (P-PSD) fitted to the product dissolution. The P-PSD was preferred over the DLM scalar since it provided a better data description in vitro and allowed the prediction of the impact of formulation on the PK profiles. P-PSDs were estimated for two batches of Phase 1 formulations and two batches of Phase 3/commercial formulations and used as inputs into the model. The PPBM generally recovered both AUC and C_max_ of acalabrutinib in the dose range of 25 to 100 mg when administrated alone in 13 arms from 5 clinical studies. In addition, the model predicted the AUC ratios of 0.68 and 0.51 in the presence of multiple doses of 40 mg omeprazole using a commercial batch of L0505009 and Phase 1 batch of NVTF, respectively. The prediction is consistent with observed AUC ratios of 0.58, 0.51, and 0.63 in three different cohorts (batch L0505009) and 0.43 (batch NVTF). Finally, the drug substance PSD of acalabrutinib was acceptable within the clinical range tested, and specifications were set based on clinical experience. The PBBM model was used to define the boundaries of the safe space for DS PSD, and simulations were bridged to all measurements of size using an equivalent sphere diameter approach.

#### 2.3.2. Case Study 2: Establishing Clinically Relevant Specifications in Pre-Approval and Post-Approval Environment for an Orally Administered Compound Formulated in Several Immediate release Drug Products (Christophe Tistaert)

This case study demonstrated how clinically relevant specifications were established for polymorphic purity and quality control dissolution for two different formulations containing JNJ-X. The compound is classified as BCS Class 4, characterised by poor solubility and intermediate permeability. Despite its classification, the compound shows significant solubilisation in the presence of bile salts, resulting in dose-linear PK across the therapeutic dose range, absence of food effect, and broad particle size range, without impact on safety or efficacy.

A new crystal form was isolated from the drug substance crude crystallisation step during commercial manufacturing. The new form appeared to be the thermodynamically favoured form in the process conditions. As the solubility of the newly isolated form was about 40% lower, compared with the registered form, and no in vivo data were available, the impact on the bioavailability of the compound was investigated by applying PBBM. Based on the available physicochemical properties, in vitro biopharmaceutical experimentation, biorelevant dissolution data, and clinical PK parameters, the model predicted the absence of any clinically relevant effects of the new form on the oral bioavailability of the drug product within the particle size specification settings and independent of the clinical dose and prandial state. In the next step, a confirmatory clinical study was performed to bridge the modelling and simulation outcome with in vivo data. The study was a single-dose, open-label, randomised, four-way crossover pivotal study in healthy adult subjects under fasted conditions, comparing the reference formulations of JNJ-X with three test formulations containing 10%, 50%, and 100% of the new crystal form, respectively, at the highest clinical dose. All formulations were found to be bioequivalent, confirming the outcome of the in vitro and in silico biopharmaceutical risk assessment.

In a next step, and as part of a continuous improvement programme, the existing PBBM model was used as the basis for assessing the potential impact of the QC dissolution profiles and, by inference, the API particle size, on the oral bioavailability of a newly developed fixed-dose combination drug product (FDCDP) containing JNJ-X. The combined use of virtual bioequivalence trials and parameter sensitivity analysis did not indicate any risks towards the bioavailability. Significant changes in the dissolution rate, well beyond the observed range, were required to result in clinically relevant changes in exposure compared with the in vivo results from the pivotal batch. Accordingly, the model indicated that the approved particle size specifications of the drug substance do not alter the oral bioavailability of JNJ-X in the FDCDP and that QC dissolution specifications comprising drug product batches manufactured within the approved particle size specifications of the drug substance are acceptable.

In both assessments, the drug product criticality analysis and available biopharmaceutics information played key roles. The significance of the highest risk critical quality attributes (CQAs) was investigated by means of in vitro, in vivo, and in silico data. The CQAs were either identified as clinically relevant, or the data were used to support the design of a safe space to ensure that the CQAs are always met.

### 2.4. Webinar 4: Overview of Global Regulatory Trends within CRDS including Progress, Challenges, and Emerging Opportunities (Aris Dokoumetzidis and Om Anand)

#### 2.4.1. Absorption PBPK Models in Regulatory Applications: The EMA Experience (Aris Dokoumetzidis)

The first part of the webinar discussed the EMA experience from the absorption of PBPK models in regulatory applications. Whilst PBPK is considered a powerful tool for in silico clinical trials, it is more difficult to validate compared with data-driven approaches such as population pharmacokinetics (PPKs). In order to accept PBPK models for high-impact regulatory applications, the EMA guideline for PBPK [23] demands thorough qualification against external datasets for the particular intended purpose, which can be a deterrent for applicants. Two case studies were presented from recent submissions to EMA containing absorption PBPK models, followed by ideas about the better uptake of such models for regulatory decisions.

The first case study shared described the provision of scientific advice for the development of a level A IVIVC for a modified release, BCS Class 2, narrow therapeutic index drug, suitable for biowaivers for post-approval variations and changing manufacturing site. The role of the PBPK model was mostly in the deconvolution step when the IVIVC guideline [22] was followed. The opinion of the assessors was, in general, positive, as the IVIVC is considered an established tool, and data from the actual product were available. In the second case study, using clinical data from a BE study of the top strength of a product, a PBPK model was used to generate a virtual bioequivalence (VBE) study aiming to waive studies for lower strengths. The assessors had a negative opinion due to the lack of qualification and less than satisfactory performance of the model and advised the sponsor to pursue an in vitro data comparison approach instead. Indeed, in a survey published by Mitra et al. [9], among applications of PBPK, modelling biowaivers based on VBE have been those associated with the highest risk. The acceptability of risks associated with the uncertainties introduced by a model should be viewed within the perspective of the potential patients’ medical benefit from the respective decision, i.e., in a context of a benefit–risk ratio. For example, models containing relatively high risks were more likely to be accepted by the authorities in paediatrics than BE studies, due to the feasibility issues often posed in conducting paediatric clinical studies and the high medical benefit for the patients. 

Steps to increase the uptake of PBPK models and, in general, modelling-and-simulation (M&S) approaches, in regulatory settings, need to include (1) systematic research to build an M&S ‘confidence space’, such that challenging research problems are already answered by the time they are reviewed by the assessors while collaborations between regulators, industry, and academia to define questions and address them systematically are needed; (2) introduction of standards for good practices and model assessment for PBPK, similarly to PPK which is more advanced in this respect, while these standards could evolve to guidelines in the future.

#### 2.4.2. Clinically Relevant Dissolution Specifications: A Biopharmaceutics Risk-Based Approach: An FDA Perspective (Om Anand)

The second part of the webinar discussed experience from the FDA, with a focus on the rationale for using a biopharmaceutics risk-based approach towards developing clinically relevant dissolution specifications to ensure patient-centric quality standards. The covered topics included a historical perspective on dissolution testing, with an emphasis on biopharmaceutic considerations for the selection of clinically relevant dissolution specifications, illustrated by a few case studies [31].

A quality product of any kind consistently meets the expectations of the user [32]. Patients expect that every dose of medicine they take be safe and effective [33]. Pharmaceutical quality assures every dose is safe and effective, and free of contamination and defects. The focus of the US FDA is to establish patient-centric quality standards [34,35]. Clinically relevant dissolution specification is defined as ‘*a specification that takes into consideration the clinical effect of variations in dissolution ensuring a consistent safety and efficacy profile*’ [10]. Establishing clinically relevant dissolution specifications aims at achieving patient-centric quality standards.

From a historical perspective, in vitro dissolution testing of drug products began with the expectation that there is a relationship between in vitro dissolution data and in vivo bioavailability of the drug [36]. In the late 1960s and early 1970s, in vitro dissolution testing became mandatory for several drug products as advances in quality-control testing and regulatory progress on in vivo relevance of dissolution continued [10,12,37]. In the past few years, a paradigm shift has occurred, and the focus has again shifted to the development of an in vitro dissolution test that provides predictive insight into the in vivo performance of the test product [10,36,37].

The US FDA takes a biopharmaceutics risk-based approach for the selection of dissolution specifications (method and acceptance criterion) for a drug product. Although the examples presented were for solid, oral dosage forms, similar principles could be applied to other dosage forms, where in vitro dissolution/release specifications are needed. The current practice in the FDA, when establishing a dissolution method for a drug product, is to determine the initial risk associated with the drug product.

Once the initial risk is determined, the information needed to mitigate the risk associated with the drug product is requested. Specifications are established to prevent any dissolution failure and ensure continuous, consistent, quality performance of the drug product [31].

The Biopharmaceutics Classification System is used as a framework to determine the risk associated with immediate-release drug products [38]. The biopharmaceutics risk assessment decision tree that is used by the FDA to evaluate the risk associated with the drug products was discussed. Five different categories of risks—namely, ‘very low’, ‘low’, ‘medium’, ‘high’, and ‘very high’—and biopharmaceutics approaches to mitigate bioavailability/bioequivalence (BA/BE) risks were highlighted. In this approach, the initial biopharmaceutics risk is determined based on solubility and permeability of the drug substance, knowledge and understanding of the critical bioavailability attributes (CBAs), in vitro dissolution performance of the drug product, and the ability to detect and control the risk. The critical bioavailability attributes (CBAs) are the formulation or process attributes that are expected to critically impact the bioavailability (absorption rate and extent) of a drug product.

By walking through the risk assessment framework, it could be both demonstrated and reiterated that for BCS Class 1 and 3 drug products, the initial risk is considered very low (if the in vitro dissolution is rapid in 500 mL of 0.1 N HCl in an aqueous medium (without surfactant)) or low (if the in vitro dissolution is not rapid in 500 mL of 0.1 N HCl in an aqueous medium (without surfactant)). For drug products containing low solubility but high permeability (drug substances with rapid dissolution in a medium within the pH range 4.5–6.8 (without surfactant)), the initial risk level is also considered low (e.g., rapidly dissolving BCS Class 2 acidic drug products).

The biopharmaceutics risk increases in cases when the solubility and or the dissolution is a rate-limiting factor in absorption, and it is necessary to understand the critical bioavailability attributes (CBAs). In the current practice of the FDA, when CBAs are clearly identified, the biopharmaceutics risk is mitigated based on the discriminability of the proposed dissolution method. In cases in which CBAs can be clearly identified, detected, and controlled, the risk level is considered ‘medium’, and risk mitigation can rely on in vitro approaches. If CBAs cannot be clearly identified, detected, and controlled, it might indicate a ‘higher’ level of biopharmaceutics risk; therefore, for risk mitigation, additional in vivo/in vitro studies may be needed to develop a biopredictive dissolution test.

The biopharmaceutics risk assessment helps in determining the type and level of information and data necessary for the biopharmaceutics risk mitigation. In cases in which the initial risk level is determined to be ‘very low’, a standard dissolution test recommended in the FDA’s Dissolution Guidance could be implemented for these drug products [39]. For ‘low’ risk products, only limited dissolution method development may be sufficient to justify the dissolution specification. For ‘medium’ risk products for which in vitro approach(es) are used to mitigate the biopharmaceutics risk, a dissolution test may be developed in a way that is able to detect meaningful changes in the identified CBA(s) and provide insight into the in vivo performance of the drug product. For products in which the biopharmaceutics risk is determined to be ‘high’, based on the available in vitro/in vivo data and/or physiologically based biopharmaceutics model (PBBM) approach, an IVIVR or IVIVC may be considered to establish patient-centric dissolution specifications. In some high-risk cases, when it may not be feasible to develop an IVIVR/IVIVC based on the available in vitro and in vivo data, the biopharmaceutics risk can be considered ‘very high’. In these cases, to support patient-centric dissolution specifications, conducting additional in vivo BA studies evaluating drug product variants may be considered to establish an IVIVR/IVIVC and clinically relevant dissolution method.

The role of biopharmaceutics, as well as various approaches that can be used to establish clinically relevant dissolution specifications (CRDSs), was then considered. Establishing patient-centric drug product quality standards is a consideration while setting up the clinically relevant dissolution specifications, and biopharmaceutics plays a critical role in achieving this objective. In general, what is available is the in vitro performance of the drug product as a dissolution profile; however, the key interest is in the in vivo performance of the drug product, i.e., systemic exposure or the plasma pharmacokinetic (PK) profile. In cases in which biopharmaceutics information can link the in vitro performance, e.g., dissolution profile, with the in vivo performance, e.g., PK profile, CRDSs can be established [9].

The application of the FDA Guidance for BCS class 1 and 3 drug products was reviewed [39]. If the dissolution is rapid or very rapid under in vitro conditions (in 500 mL of 0.1 N HCl in an aqueous medium (without surfactant)/using USP apparatus 1 (basket/100 rpm) or USP apparatus 2 (paddle/50 rpm)), then the consistent bioavailability performance can be assured, and the dissolution specifications, ensuring a rapid dissolution, may be considered clinically relevant. For medium-to-high-risk products, building a safe space [10] (bioequivalence space) is recommended to establish the CRDSs. Several approaches that can be used to build a safe space were presented and briefly discussed. Briefly, the simplest approach is having an in vitro in vivo relationship (IVIVR), a rank order in the PK profiles and dissolution profiles and using a bracketing approach (confirming acceptable in vivo performance at dissolution extremes); another approach is a traditional IVIVC approach, based on which the relationship is developed and fully validated. In some cases for which the modelling approach cannot be used, an exposure-response analysis can be considered to build a safe space and CRDS [40].

The most recent and more commonly used approach to establish a safe space is through an IVIVC or IVIVR, often using physiologically based biopharmaceutics modelling (PBBM). In a PBBM approach, a mechanistic understanding regarding absorption, gut metabolism, drug transport, and elimination is developed and applied with the drug product’s critical attributes, to predict a systemic drug exposure and virtual BA/BE. The FDA’s Draft Guidance entitled ‘*The Use of Physiologically Based Pharmacokinetic Analyses—Biopharmaceutics Applications for Oral Drug Product Development, Manufacturing Changes, and Controls Guidance for Industry*’ [10] provides the FDA’s current approach and additional details on this topic. The FDA continues to encourage sponsors/applicants to develop and use a biopredictive dissolution method in conjunction with a PBBM approach. Additionally, one of the important aspects of this draft guidance is that it provides an option of using an alternative dissolution method, i.e., a biopredictive dissolution method that can be different from the regular quality control (QC) method. Prior to the conclusion, general regulatory applications of the PBBM approach to continuously support drug product quality and reduce the regulatory burden were very briefly presented.

### 2.5. Webinar 5: Future Developments with PBBM/PBPK Software Packages (David Turner and Maxime Le Merdy)

#### 2.5.1. Virtual Bioequivalence, Mechanistic Models, and Advanced Models (David Turner)

Very often, PBPK modelling applied in a biopharmaceutic context has focussed on an average or representative subject without considering the potential population variability in PK outcomes. The subjects selected for any clinical trial represent a sample of the overall population or sub-population of interest. This means that, unless highly powered, the PK outcomes from a trial may not be representative of the relevant (sub-) population. Equally, a PBPK simulation consists of a random sample of virtual subjects that are unlikely to correspond closely to the subjects in an actual clinical study even if constrained by the limits of the clinical study design (age, gender, weight, BMI, etc.). In this regard, one of the advantages of a PBPK model is that additional virtual subjects can be simulated at zero cost, aside from costs related to computational resources. It is, therefore, standard practice with a PBPK model to run 10 or even 20 replicates of a clinical trial to assess the impact of sampling [20].

Population variability of PK outcomes can arise from the variability of a wide range of physiological parameters, including plasma protein concentrations, tissue volumes, enzyme, transporter abundances, etc. Specifically, in relation to orally dosed products, there is well-established variability in gut pH, bile salt concentrations, and residence times in the different regions of the gut, plus numerous other factors [41]. Therefore, unless a drug product has both release/dissolution and absorption rate invariant to physiological variability, the need to consider variability applies equally to the biopharmaceutic component, as it does to other disposition parameters. Furthermore, in the context of the development of CRDSs (e.g., [30,42]), and where the aim is to identify dissolution safe space, VBE analysis can or indeed should be applied to provide statistical and scientific rigour. An inherent requirement to demonstrate BE, or non-bioequivalence, using widely applied crossover studies, is to be able to handle both between-subject (population) variability and within-subject variability (BSV and WSV, respectively) of PK (FDA Guidance, 2003 and 2021, for example). WSV is also often referred to as inter-occasion variability (IOV) or intrasubject variability; Wu et al. (2021) [19] provide a discussion of the nuances of these terms.

Traditionally, WSV within a BE context has been accounted for with the addition of variability to PK parameters (C_max_, AUC), based on prior clinical studies, with the assumption that the WSV established for the reference formulation is applicable to the test formulation(s), an assumption which may be incorrect [43]. An alternative approach to post hoc addition of WSV to PK endpoints is to use PBPK modelling, whereby both BSV and WSV are applied to relevant physiological parameters (gastric emptying, pH, bile salt conc, etc.) and propagated through mechanistic PBPK models, resulting in BSV and WSV of simulated PK when using appropriate study designs. With more complex mechanistic models, the variability of simulated physiological parameters may be derived from multiple other contributory physiological parameters with known BSV and, in some cases, WSV. For example, the advanced dynamic bile salt model (ADBSM) [44] integrates hepatic secretion of bile salts (BS), gallbladder filling and emptying kinetics, the phases for false conditions of interdigestive migrating motor complex (IMMC), and meal intake times to predict time concentration profiles of luminal BS. The simulated BS concentrations are also dependent on luminal fluid volumes and their time course and regional variability. Thus, simulated bile salt concentrations vary with time, have BSV and WSV, and require that solubility be recalculated dynamically during the simulations.

This novel approach to deriving WSV (and BSV) of PK endpoints from the underlying physiology requires knowledge of the WSV and BSV of relevant physiological parameters. While there is some information in the literature on BSV, this is limited and a major gap in the effective application of VBE tools is information on WSV. Bego et al. (2021) [43] provide a rigorous discussion of the issues related to VBE and suggest an interesting model-based approach to estimating WSV of physiological values from multiple clinical datasets.

An essential prerequisite for the application of PBPK-based VBE is that suitable mechanistic models are available with sensitivity to the relevant physiological variabilities (WSV-BSV). With respect to capturing the variability of drug product dissolution, this requirement precludes the direct input of in vitro dissolution profiles into a PBPK model. This statement applies regardless of the biorelevance or biopredictivity of an in vitro profile because such a profile can only represent in vivo dissolution for a single subject, usually a representative or average subject. Of course, variability can be added to such profiles in various ways, such as adding a CV% to measured data points, but the question that then arises is ‘what should these variabilities be?’ The argument is the same (1) even if separate in vitro profiles are available for gastric, small intestinal, and, relevant to some drug products, colonic dissolution, and (2) even where an absorptive component is mimicked in vitro, such as with a biphasic dissolution [45], or more complex experiments such as the TNO TIM-1 (The TIM Company, Delft, The Netherlands) or the DIAMOD (ProDigest, Gent, Belgium) approaches.

There are further limitations and assumptions required when using in vitro dissolution as a direct surrogate for in vivo dissolution, not least the fluid volume that is applied, which, if insufficient, may not completely dissolve the drug product (where the API may be fully dissolved in vivo) but which, if too large, may overestimate the in vivo dissolution rate. For example, Purohit et al. (2018) [46] performed in vitro dissolution experiments with the poorly soluble, non-ionising drug tacrolimus. Experiments were performed using a compendial medium at four different static fluid volumes (40, 100, 450, and 900 mL). A question then arises, ‘which of these in vitro profiles should be used as input to a PBPK model?’ More generally, depending upon the drug BCS class and dose, some of these caveats may not apply. Having said that, if the very high BSV of fluid volumes picked up through MRI studies of the gut (which is the case, for instance, in the study of Mudie et al. (2014) [47]) truly represents in vivo conditions, then assessing drug product dissolution using simple rules of thumb such as the BCS may be irrelevant for at least some subjects in a clinical study.

**Figure 2:**
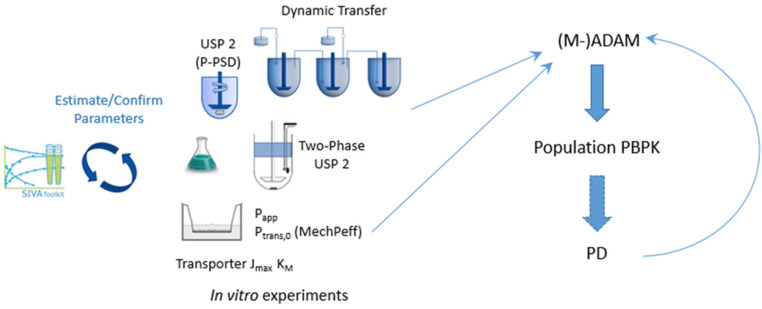
Coupling modelling of in vitro experiments to PBPK models. A workflow for PBPK modelling: biopharmaceutic IVIVE.

One approach to address some of the issues raised above is to apply mechanistic models of dissolution for which suitable input parameters for these sometimes-complex models are required. These can be obtained with direct experimental measurement and/or can be estimated from the modelling of suitable in vitro experiments (Figure 2) [48,49,50]. In cases in which, for the same drug product batch, multiple in vitro dissolution profiles are available, measured under different conditions (pH, surfactant concentration, rpm, fluid volume, etc.), then either simultaneous fitting and/or separation of the data into training and test sets can add confidence to the model estimates. Overall, this approach has the advantage that confidence can be gained in intrinsic input parameters (intrinsic solubility, pKa, Ksp, bile micelle partition coefficients, mono or polydisperse PSD, etc.) prior to their use in a PBPK model. This, in turn, (1) reduces or eliminates the need for estimation (adjustment) of these parameters within the PBPK model where, due to a large number of adjustable parameters, parameter identifiability is usually an issue, and (2) means the user can focus attention on alternative parameters or assumptions of the model where simulations do not match clinical observations. In this regard, and with appropriate tools such as SIVA, useful inputs for PBPK models can be obtained from non-biopredictive experiments such as QC dissolution, in which hydrodynamics, fluid volumes, and other factors are significantly different from in vivo conditions. Pepin et al. (2021) [51] describe algorithms for factoring out these differences so that they are not propagated into the PBPK models—the fluid hydrodynamics components described have been available in the SIVA Toolkit for some time (e.g., Pathak et al. (2017) [48]).

For an average subject, it may be that a mechanistic dissolution model provides the same or very similar in vivo dissolution (and, therefore, PK outcomes) as a model where an in vitro profile is directly input to the PBPK model. However, the latter provides no way to account for the population variability of dissolution, which is linked to physiological variability. For some API/drug products, this variability may not be significant, but in general, the possibility should be addressed. Additionally, as with PBPK models in general, a mechanistic model allows extrapolation to different conditions which may arise in certain disease populations. A more subtle point that may be relevant for certain drug products is that the small intestine is not a homogeneous environment, and there are regional differences in pH, bile salt concentrations, and fluid volumes such that for susceptible products dissolution rate may be significantly different in these different environments. A mechanistic model of dissolution, even accepting it is not perfect, will be sensitive to these differences.

In conclusion, appropriately parameterised mechanistic models of drug product release and dissolution, coupled with detailed knowledge of relevant physiological parameters, provide the way forward for more realistic simulations of dissolution accounting for population variability—namely, BSV, WSV, or extrapolation to different populations; VBE analysis requires accounting for both BSV and WSV. A current limitation of the biopharmaceutic IVIVE approach is that mechanistic models are available to capture dissolution of particulate IR formulations but are somewhat limited where the disintegration of these formulations is rate-limiting, with regard to handling modified/controlled release products and for enabling formulations such as amorphous solid dispersions [6]. There are a number of empirical/semi-mechanistic approaches available [52,53], but these, in general, cannot be extrapolated to variable conditions in vivo, or only to a limited extent. Thus, at least part of the future of PBPK/PBBM lies in the development of mechanistic models able to capture drug product disintegration (IR) and, for enabling formulations and MR and CR formulations, release, and dissolution. These models then need to be coupled with tools to make the translation to in vivo via appropriate IVIVE approaches. Concomitant to the models is the requirement for descriptions of the variability of relevant in vivo physiological parameters, both between- and, where relevant, within-subject variability. A key component of this is the need to capture covariation of physiological parameters relevant to biopharmaceutics. A step in this direction is provided by complex models that simulate physiological parameters and their variabilities from the known variabilities of other parameters, an example of which is the ADBSM described above.

#### 2.5.2. How to Use Modelling and Simulation to Link In Vitro Dissolution to Drugs In Vivo Behaviour (Maxime Le Merdy)

The modelling-and-simulation (M&S) technique is a continuously evolving interdisciplinary specialty and is an intrinsic component of drug product research and development programmes. PBBM/PBPK models have multiple applications with different associated risks, depending on their purpose. The industry and some regulatory agencies view the prediction of pH-related drug–drug interactions (DDIs) as low risk, whereas waiving human PK clinical evaluation for major formulation changes using virtual bioequivalence (BE) is considered high risk [8]. The PBBM approach presents numerous advantages, as it provides mechanistic insight into formulation behaviour within the gut lumen; however, some enhancements to the tools are still needed for its straightforward utilisation in the drug product quality specific applications (e.g., integrate formulation disintegration, swelling, etc.) [21]. GastroPlus^®^ is a PBBM/PBPK mechanistic-based simulation software package and is largely used to predict in vivo behaviour of oral and non-oral drug products based on in vitro dissolution data. A recent survey among users within the generic drug industry showed that GastroPlus has been used in regulatory submissions to widen dissolution acceptance criteria and other drug product specifications, so as to support major CMC changes and/or propose alternative approaches to demonstrate BE to fulfill 505J requirements. To meet those requirements, relationships between in vitro assays and in vivo PK exposure are necessary.

**Figure 3:**
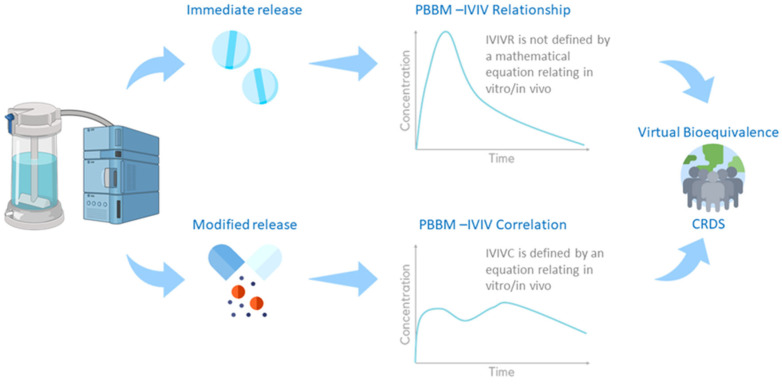
Framework for virtual bioequivalence and CRDSs.

As part of the drug product development process, extensive information is generated to characterise the critical quality attributes (CQAs) and their impact on the in vitro (e.g., dissolution profiles) and in vivo performance (e.g., plasma concentration (Cp) time course) in preclinical and clinical settings. The current typical paths for PBBM-based predictions of in vivo clinical Cp-time profiles using in vitro data are partly determined by the dosage form. For immediate-release (IR) formulations, in vitro to in vivo relationships (IVIVRs) are usually developed. For modified-release (MR) formulations, in vitro to in vivo correlations (IVIVCs) are typically generated. The IVIVCs are defined by an equation relating a drug product in vitro release/dissolution to its in vivo pharmacokinetics, contrary to IVIVRs. Once the IVIVRs and IVIVCs are validated, they can be used to predict the in vivo PK of formulation variants using in vitro dissolution/release observations. Clinically relevant dissolution specifications (CRDSs) can then be established based on virtual BE analysis (Figure 3).

To create and validate an IVIVR using PBBM, in vitro dissolution data are utilised to parameterise the model to predict the API in vivo dissolution. In GastroPlus, the Johnson [54] or Z-factor [55] dissolution models are typically utilised to model the IR formulation’s in vivo dissolution. The first method fits a particle size distribution (PSD) using in vitro dissolution profiles. The fitted PSD is then used as an input in the Johnson model to predict the in vivo dissolution. This approach, known as the P-PSD approach, was extensively described in other presentations during this workshop, as well as in the literature [56,57]. The second method consists of fitting the Z-factor parameter based on in vitro dissolution profiles. The fitted Z factor can then be used to calibrate the Z-factor model to predict drug products in vivo dissolution.

The following two examples highlight how the approaches have been used successfully:

(1) The Z-factor method has been used by the generic industry to (1) establish an IVIVR; (2) predict in vivo PK profiles of test and reference products based on their in vitro dissolution profiles and the validated IVIVR; (3) use virtual BE trials to predict the BE between the test and reference formulations for a BCS Class 2 compound [58].
(2) The US FDA used an IVIVR to investigate the clinical impact of potential changes in warfarin crystalline form upon storage in different conditions. The IVIVR predicted PK profiles could be perfectly overlaid with BE clinical trial results, demonstrating the great predictive ability of this M&S virtual BE method [59].

IVIVCs have been created using either traditional methods or PBBM/PBPK mechanistic methods. For the latter, to successfully establish an IVIVC, the baseline model must be validated using intravenous (IV) and/or IR datasets. The validated model is then used to deconvolute the in vivo dissolution/release profile using the mechanistic IVIVC method in GastroPlus. This process consists of fitting formulation-specific Weibull function parameters to capture the observed PK profiles for corresponding MR formulations. Finally, a Level A correlation is generated between all the predicted in vivo dissolution/release profiles and the corresponding observed in vitro release profiles. This correlation provides the mathematical equation characteristic of IVIVCs. The validation criteria for an IVIVC have been defined by the US FDA Guidance [10,37]. Once validated, the IVIVC can be used to predict clinical drug product PK profiles based on in vitro dissolution only. A pharmaceutical industry perspective on establishing IVIVCs using PBBM/PBPK models was recently published [60]. Kesisoglou et al. compared traditional and mechanistic IVIVCs for MR formulation of a BCS Class 3 compound and demonstrated the superiority of the mechanistic method [61].

Ultimately, once an IVIVR/IVIVC is established and validated, it can be used to define the clinically relevant dissolution specification of an IR/MR formulation by simulating in vivo PK profiles of formulation variants based on in vitro dissolution/release profiles. The simulated PK profiles can then be compared by running virtual BE studies which integrate both inter-subject and intra-subject variability.

### 2.6. Webinar 6: Emerging Opportunities within PBPK/PBBM Modelling to Support CRDSs, including New Research Areas (Adam Darwich, Brendan Griffin, and Jennifer Dressman)

#### 2.6.1. Absorption Modelling: A Brief History, Emerging Trends, and Path Forward (Adam Darwich)

Since the inception of PBPK absorption models in the early 1980s [62,63,64], these models have evolved together with the biopharmaceutics discipline. Systems modelling has been valuable in integrating the many sources of information that together make up our understanding of oral bioavailability and drug exposure. This has allowed the study of ‘known knowns’ and ‘known unknowns’ and defined future research directions.

Recent advances in biopharmaceutics and related fields reinforce the relevance of the integrated systems approach. In vivo data on fluid dynamics, proteomics, transcriptomics, and drug behaviour can improve our ability to characterise variability in drug exposure through the use of systems modelling [65,66,67,68]. New in vitro experimental techniques, coupled with modelling, improve the translation of formulation effects from in vitro to human subjects [69]. Middle-out modelling approaches allow the estimation of variability and reverse translation of physiological parameters, leading to opportunities in virtual bioequivalence and mechanistic in vitro–in vivo extrapolation of drug formulations [70,71,72,73]. Real-world data (RWD) from healthcare and registries are combined with PBPK to inform drug development in special populations and derive inference from pharmacovigilance data [74,75]. Further, the wider adoption of advanced simulation methods in pharmaceutics modelling, such as global sensitivity analysis, allows more systematic interrogation of the impact of information on simulation outputs. This is to inform model development and generation of data [76,77].

Advances in PBPK absorption modelling rely heavily on the development of the biopharmaceutics discipline. The increasing body of knowledge opens new opportunities for utility. PBPK and other modelling methods can facilitate the integration of novel data by providing a systems approach to information and inference. New, combined modelling approaches are necessary to accommodate inference of novel data to the field, such as RWD.

#### 2.6.2. Predicting Preclinical Outcomes: An In Vitro–In Silico Approach to Guide Oral Formulation Design (Brendan Griffin)

With estimates of >75% of new drugs emerging from drug discovery programmes displaying poor solubility, there is a need to develop novel bioenabling technology for efficient oral delivery [78]. With the industry increasingly operating with accelerated drug product development timelines, there is also a need to ensure formulation optimisation is completed as early as possible in development and crucially prior to pivotal proof of concept clinical trials. While preclinical studies play key roles in exploring oral pharmacokinetics/toxicology and informing formulation selection, there is also an opportunity to utilise in silico tools to predict formulation performance in preclinical models. Strategies to advance the application of species-specific in vitro and in silico models for predicting preclinical oral pharmacokinetics can streamline drug product developability, improve preclinical to clinical translation, and reduce overall requirements for preclinical in vivo data.

Funded under the Horizon 2020 Marie Sklodowska-Curie actions programme, the PEARRL (www.pearrl.eu) Network, brought together the European pharma industry, academia, and regulatory agency partners in a multisectoral team focused on developing innovative oral drug development strategies, tailored to facilitate accelerated drug development timelines [79]. Working within the PEARRL academic–industry collaboration, scientists from University College Cork, National and Kapodistrian University of Athens, Goethe University in Frankfurt, and Janssen Research and Development, Belgium, explored the suitability of the pig model as a preclinical model for predicting oral bioavailability in humans [80]. Research showed that correlations between drug bioavailability in pigs vs. humans (*n* = 20) were comparable to those previously reported for dogs vs. humans [81] and demonstrated the suitability of the pig as a preclinical model to predict human oral drug absorption. A comprehensive characterisation of gastrointestinal (GIT) conditions of landrace pig models was performed using a telemetric motility capsule (SmartPill^®^) to assess GIT conditions, under fasted and postprandial conditions [82]. This led to the establishment of a biorelevant medium to mimic porcine intestinal fluid, i.e., porcine fasted state stimulated intestinal fluid (FaSSIFp) [83]. This study demonstrated that FaSSIFp was superior at predicting the solubility of the six model drugs, compared with human simulated intestinal fluid (FaSSIF), and confirmed that species-specific intestinal media provide more accurate predictions of biopharmaceutical properties in preclinical models. Additionally, the availability of a FaSSIFp offered the next step in advancing an integrated in vitro–in silico approach to predict in vivo absorption in pigs [84]. By combing porcine-specific biorelevant in vitro parameters with a porcine physiologically based pharmacokinetic (PBPK) model that considers the species-specific GIT transit conditions of landrace pigs, this novel approach allowed prospective prediction of the drug absorption profiles in a preclinical setting. The study demonstrated that the combined in vitro–in silico porcine model reliably predicted the experimentally observed plasma concentration profile under fed state conditions in pigs and, therefore, offers an improved approach for predicting the impact of food on dosage form performance in a preclinical in vivo setting. In summary, by establishing an in vitro biorelevant intestinal media model and an in silico PBPK model that collectively capture the intestinal absorptive conditions of the specific animal model, this integrated in vitro–in silico approach can support guidance in early decisions of drug product development, rationalise animal model selection and reduce the overall number of animals needed in oral drug product development testing.

#### 2.6.3. An In Vitro–In Silico Approach to Predicting Oral Absorption of Drugs When Co-Administered with PPIs (Jennifer Dressman)

In recent years, both the pharmaceutical industry and the regulatory authorities have become increasingly interested in mechanistically understanding and potentially predicting how various dosing situations will affect the pharmacokinetic response to oral drug administration. Although food effects have been studied extensively in terms of understanding the GI physiology in the fed state better [85,86,87,88] and designing dissolution release tests to predict these [89,90,91,92,93], other ‘co-administration’ events have received less attention.

In a collaboration between AstraZeneca scientists and the Goethe University in Frankfurt, biorelevant media have been developed to help understand the absorption of drugs when they are co-administered with proton pump inhibitors (PPIs). A review of the physiological changes in response to PPI administration revealed a wide spectrum of gastric pH responses, depending on the potency and dose of the PPI administered. For this reason, media were developed to bracket the behaviour across the spectrum, with media composed at pH 4 and 6 [94]. Although gastric volumes are also lower during PPI therapy due to reduced gastric acid output, a volume of 250 mL was deemed to adequately capture the conditions in the stomach when a glass of water is ingested with the dosage form. An important aspect of the media design was to reflect the low buffer capacity of the gastric contents in the (relative) absence of gastric acid, as this means that the drug and/or the formulation excipients can potentially influence the gastric pH.

Three examples (two weak bases and one weak acid) were used to illustrate how combining the dissolution results with Simcyp software was able to successfully bracket the plasma profile with and without co-administration of PPIs. The first was a poorly soluble weak base in development at AstraZeneca. This compound enjoyed a data-rich scenario, enabling the post-absorptive PK to be based on IV data and ample studies to enable both the setup and validation of the oral absorption model in Simcyp^®^. Applying the dissolution results in the PPI media, it was possible to bracket the PK response in two studies in which PPIs were co-administered with the drug [95]. An analogous approach was successfully applied to another poorly soluble weak base, dipyridamole, even though all PK data had to be retrieved from external sources [96]. Finally, the media were used to explore the possibility that precipitation of a weak acid in the stomach after administering a salt form could be ameliorated by co-administration of PPIs. The weak acid used in these studies was raltegravir, which is available commercially as potassium salt.

## 3. Q&A Sessions—Key Themes

During each webinar, delegates were provided with an opportunity to discuss the information and content shared. Each of the six webinars generated a wide range of questions. The key themes from across the series fall into three main categories and are highlighted below in Table 1. 

## 4. Conclusions

The webinar series provided opportunities for industrial, academic, and regulatory scientists to further discuss the topic of developing clinically relevant dissolution specifications (CRDSs) for oral drug products. The series heard from industrial scientists on how the thinking within the CRDS space is evolving, and how the specific approach of PBBM is being utilised, as illustrated with case examples. This had been defined as an action topic from the 2017 APS One Day meeting [6]. Perspectives from regulatory agencies and their experiences thus far were also shared during the series. Finally, emerging opportunities within PBPK/PBBM modelling to support CRDSs and drug product development were discussed, both by the speakers and in the Q&A sessions.

Although it is clear from the presentations and Q&A sessions that we continue to make significant progress in the field of CRDSs and the utility/success of PBBM, there is also a need to continue the momentum and dialogue between the industry and regulators. The following five key areas which require further discussion and harmonisation were identified:

- PBBM modelling approaches and their utility, including model verification and validation. Further dialogue is required to clearly understand requirements and manage expectations both for the industry and regulators;
- Role of dissolution data (QC or more biorelevant media) as appropriate input into PBBM models;
- Clinical study design to support the setting of clinically relevant dissolution specifications;
- Regulators and the industry should develop a CRDS roadmap and framework for implementing CRDSs;
- Opportunity to engage and set up an EMA–Industry workgroup on CRDSs and PBBM in pharmaceutical applications.

## Figures and Tables

**Table 1:** Webinar Q&A Sessions: Major Discussion Topics and Resulting Themes.

Strategy and Data Input	Design of Studies (Clinical or Preclinical)	Data Utilisation/Regulatory Impact
Understand/define which CQAs/CBAs can be explored and/or mitigated using PBBM	Understand/define which CQAs/CBAs ones to test in clinical studies (relBA or BE?)	Understanding CQAs/CBAs is critical to putting together a well-designed biopharmaceutical risk assessment
A biorelevant method is not necessarily clinically relevant until a link to in vivo performance has been shown. Can a biorelevant method ever make it to QC status? Could this be BCS/DCS driven?	In vivo clinical study setup to claim a dissolution safe space relBA or BE study? 80–125% with 90% CI or GMR? What limits could be acceptable to set specifications as the drug product variants will not be the commercial drug product?	Use of the terms biorelevant/clinically relevant; is the terminology consistent yet? If and how are biorelevant dissolution methods used in the development space and how can this information be shared at the IMPD or market application stage to enable efficient life cycle management?
Ways of including dissolution data into PBBM models: Z factor or API PSD/P-PSD and success rates of both? Will the input depend on the question? Use of PBDT data or QC data in PBBM models—should we approach this depending on BCS/DCS? BCS 1/3 could use QC, however highly likely a model is not required for regulatory specs. Use biowaiver guidelines? BCS 2/4 harder due to % surfactant required in QC release method, could we use PBDT? For BCS2/4 IR formulations include mechanistic modelling of dissolution?	Utilisation of totality of clinical relBA/BE type studies in the model verification step, together with CBA/CQA specifically designed studies Justifying studies in companies that share accountability for product quality across divisions is easier than for companies where development and manufacturing are disconnected—would industry agree?	Interaction with agencies to discuss compound strategy advocated. Experience is time to submit/receive feedback is quite lengthy. Can we look at how to improve this to encourage more interactions? Industry examples demonstrate that CRDS/PBBM modelling approaches are being used with some success.
Model should be fit for purpose/build to address the question. Are we clear on the level of model validation expectations? Modelling variability (i.e., which factors to include, e.g., gastric emptying, stomach pH, transit time); Validation set including acceptable prediction error; Requirement for non-BE batches.	In which circumstances can we use the models in place of clinical trials, or will the models only be accepted for specification setting and post-approval changes?	Guidance on model setup vs. model verification/validation essential Differences in global regulatory acceptance of mechanistic absorption models in specification setting, IVIVC acceptance compared with IVIVR acceptance; agencies continue to learn together with industry
Understand sources of variability To investigate In vivo API and DP performance	Mechanistic Modelling is a key area of growth.	Reduce the number of animal experiments when it is clear that the best model for humans is human; learn more about drug–drug interactions at the level of absorption (i.e., PPIs), as this can impact labelling/dosing times.

## Abbreviations

ADAM	Advanced dissolution absorption and metabolism
ADME	Absorption distribution metabolism excretion
ADBSM	Advanced dynamic bile salt model
API	Active pharmaceutical ingredient
APS	Academy of Pharmaceutical Science
AUC	Area under the curve
BA	Bioavailability
BE	Bioequivalent
BCS	Biopharmaceutical Classification System
BioRAM	Biopharmaceutics risk assessment roadmap
BMI	Body mass index
BS	Bile salts
BSV	Between subject variability
CBA	Critical bioavailability attribute
CI	Confidence interval
CMC	Chemical and Manufacturing Controls
CMA	Critical material attributes
Cmax	Maximum concentration
Cp	Plasma concentration time profile
CPP	Critical process parameters
CQA	Critical quality attribute
CRDS	Clinically relevant dissolution specifications
CR	Controlled release
CV	Coefficient of Variation
DCS	Development classification system
DDI	Drug–drug interaction
DLM	Diffusion layer model
DP	Drug product
DS	Drug substance
EMA	European Medicines Agency
EU	European Union
FaSSIF	Fasted state simulated intestinal fluid
FaSSIPp	Porcine fasted state simulated intestinal fluid
FDA	Food and Drug Administration
FDCPD	Fixed-dose combination drug product
GI	Gastrointestinal
GIT	Gastrointestinal tract
GMR	Geometric mean ratio
HCl	Hydrochloric acid
HPLC	High-pressure liquid chromatography
ICH	International Committee for Harmonisation
IMMC	Interdigestive migrating motor complex
IMPD	Investigational medicinal products
IVO	Inter occasion variability
IR	Immediate release
IV	Intravenous
IVIVE	In vitro–in vivo extrapolation
IVIVC	In vitro–in vivo correlation
IVIVR	In vitro–in vivo relationship
MR	Modified release
MRI	Magnetic resonance imaging
M&S	Modelling and simulation
NDA	New drug application
PhEUR	European Pharmacopeia
PBDT	Physiologically based dissolution testing
PBPK	Physiologically based pharmacokinetics
PBBM	Physiologically based biopharmaceutics modelling
PK	Pharmacokinetics
PPI	Proton pump inhibitor
PPK	Population pharmacokinetics
P-PSD	Product particle size distribution
PSD	Particle size distribution
QC	Quality control
RWD	Real-world data
SIF	Simulated intestinal fluid
SGF	Simulated gastric fluid
SUPAC	Scale-up and post-approval changes
USP	US Pharmacopeia
VBE	Virtual bioequivalence
WSV	Within-subject variability
